# Optimization and Detection of Freshness Biomarkers of Atlantic Salmon Subjected to Different Vacuum Packaging Conditions during Storage at 0 °C by Metabolomics and Molecular Docking

**DOI:** 10.3390/foods13172714

**Published:** 2024-08-27

**Authors:** Yu-Shun Lu, Gui-Xiao Yao, Jiang Yu, Jing Qiu, Yong-Zhong Qian, Xuan-Yun Huang, Yan-Yang Xu

**Affiliations:** 1 Key Laboratory of Agro-Product Quality and Safety, Institute of Quality Standards and Testing Technology for Agro-Products, Chinese Academy of Agricultural Sciences, Beijing 100081, China; 2 Institute of Special Animal and Plant Sciences, Chinese Academy of Agricultural Sciences, Changchun 130112, China; 3 Faculty of Printing and Packaging and Digital Media, Xi’an University of Technology, Xi’an 710048, China; 4 East China Sea Fisheries Research Institute, Chinese Academy of Fishery Sciences, Shanghai 200090, China

**Keywords:** Atlantic salmon, vacuum packaging, flavor nutrient, storage time, TIR1/TIR3

## Abstract

The freshness of Atlantic salmon is influenced mainly by tissue metabolism, which in turn is affected by storage time and conditions. The alterations in taste profiles and nutritional values of salmon when packaged using vacuum methods have not been fully understood, and the factors contributing to these changes require further research. In this work, the extraction method for flavor nutrients from salmon was optimized via the Plackett–Burman (PB) test. A sensitive and rapid targeted metabolomics method for the simultaneous determination of 34 nutrients was successfully established via ultra-performance liquid chromatography–triple quadrupole/linear ion trap composite mass spectrometry (UHPLC-QTRAP/MS), and various nutritional compositions during storage at 0 °C under different vacuum conditions (0 kPa or −90 kPa) for 4 and 8 days were analyzed. Results showed that storage time had a significant effect on salmon metabolism. The total amino acids decreased by 62.95% and 65.89% at 0 kPa and −90 kPa, respectively. Notably, a marked reduction in histidine after 8 days at −90 kPa may have diminished bitterness, while decreased levels of umami-tasting amino acids like glutamine and aspartic acid affected the overall flavor profile. Overall, the packaging conditions at 0 °C and 0 kPa were more suitable for the preservation of most nutrients in salmon. Pathway enrichment analysis revealed that the reduction in substances was mainly related to the alanine, aspartate, and glutamate metabolism pathways. Alanine, inosine, and histidine, whose levels changed significantly, can bind to the typical umami taste receptor TIR1/TIR3 and can be biomarkers to monitor and determine the freshness or spoilage of salmon after 4–8 days of storage. This study revealed the changes in small-molecule nutrients in salmon during storage under different packaging conditions, which provides a reference for the packaging preservation technology of fresh salmon and new ideas for the evaluation of salmon quality and determination of freshness.

## 1. Introduction

Atlantic salmon is one of the most traded and consumed aquatic products worldwide because of its high nutritional value. Atlantic salmon is known for its bright red–orange flesh and deep, rich flavor; it contains a variety of high-quality animal proteins and essential amino acids [1] and is easy to digest and absorb by the human body [2]. It is also a good source of n-3 polyunsaturated fatty acids (n-3 PUFAs), fat-soluble vitamins, trace elements, and other important biologically active substances for humans [3,4,5,6]. These nutrients can promote growth and development [7], reduce cholesterol levels, and have important functions that prevent and treat cardiovascular and cerebrovascular diseases [8,9], diabetes, and cancer [10]. However, salmon is perishable during storage due to its smaller muscle fiber dimensions but higher moisture content compared to other fish; salmon quality degradation during storage is caused by different damage mechanisms, such as microbial activities [11], autolytic enzymatic reactions, lipid oxidation [12], and pathogenic microorganisms [13]. The taste, nutritional content, and safety of salmon are crucial for both public health and consumer preference, as well as for the global seafood industry [14]. Therefore, developing reliable methods for the evaluation of salmon quality is highly important.

Atlantic salmon are highly perishable, and temperature is highly important in determining their shelf life. Freshness is one of the most important attributes when Atlantic salmon quality is assessed. A wide variety of chemical and physical methods have been used to assess the freshness of fish during storage, including sensory evaluation [15], microbial inspection [16], and physical and chemical detection [17]. Jääskeläinen et al. [18] evaluated the microbial changes in raw salmon and tuna fillets stored under vacuum at 3 °C over a period of 12 days and reported that trimethylamine was the main spoilage product, and that the bacterial colony count reached an average of 7.3 lg cfu/g in salmon. Moreover, the concentration of glucose decreased, and the concentration of organic acids increased during storage. Physical and chemical detection methods evaluate the quality of fish meat by analyzing the stiffness index, total volatile base nitrogen (TVB-N) value, K value, drip loss, water holding capacity, protein content, fatty acid content, and other indicators [16,19,20,21]. Pan and Lin [21] analyzed the TVB-N value, color difference value, K value, and other indicators of Atlantic salmon at -1.8 °C and 4 °C and reported that the ice temperature (−1.8 °C) preservation method can extend the shelf life by 3 days. However, few studies have evaluated the fresh quality of salmon on the basis of changes in small-molecule nutrients. Fidalgo et al. [22] analyzed the fatty acid and polyethylene indices of vacuum-packaged fresh Atlantic salmon loins after hyperbaric storage at low temperature via GC–MS and reported that hyperbaric storage samples retained fresh-like alcohols and aldehydes. The nutrient components of salmon are complex and diverse, except for fatty acids, alcohols, and aldehydes. The spoilage process caused by microbial activity produces many low molecular weight metabolites [23]. This study aimed to identify comprehensive changes in small-molecule metabolites that quantitatively indicate the degree of freshness of Atlantic salmon.

Metabolomics encompasses the qualitative, quantitative, and dynamic study of all endogenous small molecules within organs and tissues [24]. Metabolomics has been widely used in screening biomarkers related to the freshness of pork patties [25], chicken [23], eggs [26], yellowfin tuna [18], shucked mussels [27], and soybean sprouts [28]. Kritikos, Aska, Ekonomou, Mallouchos, Parlapani, Haroutounian, and Boziaris [19] researched the evolution of volatile organic molecules during the storage of European seabass (Dikentrarchus labrax) fillets and Atlantic salmon (Salmo salar) slices under modified atmosphere packaging at 2 °C and monitored total volatile basic nitrogen (TVB-N), microbiological, and sensory changes. The results revealed that the volatile organic molecules 3-hydroxy-2-butanone, 2,3-butanediol, 2,3-butanedione, and acetic acid could be potential spoilage markers. Free amino acids can be produced as a result of fish muscle protein autolysis, leading to fish muscle deterioration, which is a result of microbial growth and biogenic amine production [29]. The levels of adenine nucleotides inosine and hypoxanthine are also commonly used as indicators of muscle freshness in fish [30].

However, little research has been conducted on evaluating the freshness of salmon by small-molecule metabolites (amino acids, nucleosides, and vitamins) via targeted metabolomics. The aim of this study was to investigate the metabolite changes in Atlantic salmon stored at 0 °C under different vacuum packaging (0 kPa or −90 kPa) conditions and to screen for biomarkers of freshness via targeted metabolomics and molecular docking. This study provides more comprehensive information on salmon storage and freshness packaging by measuring the low molecular weight nutrient (amino acid, vitamin, and nucleoside) content of salmon, which provides a reference for packaging preservation technology for fresh salmon and new ideas for the evaluation of salmon quality and determination of freshness.

## 2. Materials and Methods

### 2.1. Chemicals and Reagents

The standards of 34 metabolites, including arginine (Arg), methionine (Met), glycine (Gly), valine (Val), tryptophan (Try), histidine (His), cysteine (Cys), serine (Ser), leucine (Leu), isoleucine (Iso) phenylalanine (Phe), threonine (Thr), alanine (Ala), proline (Pro), lysine (Lys), glutamine (Glu), glutamate (Gln), tyrosine (Tyr), aspartic acid (Asp), asparagine (Asn), γ-aminobutyric acid (GABA), pyroglutamic acid (Pyr), taurine (Tau), thiamine (VB_1_), riboflavin (VB_2_), nicotinic acid (VB_3_), folic acid, lumichrome, cytosine, 2-hydroxyadenine, guanosine hydrate, inosine, cytidine, 1-methyladenosine, and 2′-deoxyuridine, were purchased from Alta Scientific Co., Ltd. (Tianjin, China) and J&K Scientific Co., Ltd. (Beijing, China). MS-grade methanol and acetonitrile were purchased from Merck (Darmstadt, Germany). Water was purified with a Milli-Q system (Millipore, France).

### 2.2. Salmon Samples and Storage Conditions

Fresh salmon samples were collected from local markets in Beijing. The salmon weighed between 3 and 4 kg, and were slaughtered before sexual maturity. After slaughtering, the salmon were gutted and rinsed in running water for 30 min, followed by chilling to 0 °C in slush ice. The entire process adheres to the cold chain standards, ensuring the product is delivered fresh to the supermarket. Once there, the salmon is cut and labeled as ready-to-eat. The salmon purchased for this study were all fresh, from the same batch, and brought back to the laboratory on ice. They were then cleaned with ice water and low-temperature sterile water. Approximately 15 g (±0.5) of a salmon slice was vacuum packaged in sterile plastic nylon packaging bags at 0 kPa and −90 kPa. All salmon samples were stored at 0 °C for 8 days, and analyses were performed every 4 days. The experimental conditions are shown in Table S1. Three replications were set for all batches.

### 2.3. Extraction Method Optimized by the PB Experimental Design

The extraction solvent was optimized via single-factor experiments, and the extraction results were obtained from three typical extraction solvents: acetonitrile, water (containing 1% formic acid), and methanol/water (1:1). A Plackett–Burman factorial design experiment with 12 runs was designed to identify the significant parameters affecting the extraction of 36 nutrients. Parameters such as extraction solvent volume (V), vortex time (t_1_), ultrasonic time (t_2_), ultrasonic temperature (T), and ultrasonic intensity (E) were investigated via variance analysis to analyze the systematic error affecting extraction efficiency. The PB design experiment is shown in Table S2.

A 0.2 g homogenized salmon sample was weighed and transferred to a 5 mL centrifuge tube. After 2 mL of extraction solvent (methanol/water = 1:1) was added, the sample was mixed for 3 min via a vortex mixer to disperse the sample. Afterward, the salmon samples were sonicated for 10 min at 20 °C and 60 W and centrifuged at 10,000 rpm for 10 min at 4 °C. Subsequently, 1 mL of the supernatant was filtered through a 0.22 μm membrane filter and placed in vials at 4 °C until analysis. Quality control (QC) samples were prepared by mixing equal aliquots of the supernatants from all of the samples.

### 2.4. Metabolite Analysis via UHPLC-QTRAP/MS

The analyses of nutrients and metabolites were performed via an ultra-performance liquid chromatography–triple quadrupole/linear ion trap composite mass spectrometer (UPLC-QTrap 6500, AB SCIEX, Redwood City, CA, USA) with an Xbridge C18 column (4.6 mm × 150 mm, 3.5 µm, Waters, Milford, MA, USA). Mobile phase A consisted of formic acid/water (%), and mobile phase B consisted of formic acid/acetonitrile (%). Separation was carried out with an elution gradient. The injection volume was 2 μL, and the flow rate was set constant at 500 μL/min. The mass spectrometry conditions were set as follows: MRM-ESI scanning mode, atomizing gas pressure (Gas 1), 55 psi; heating auxiliary gas pressure (Gas 2), 55 psi; curtain gas, 30 psi; ion spray voltage floating (ISVF), 4.5 kV; and ion source temperature, 600 °C. Mass spectrometry was performed via electrospray ionization in positive ion mode, and the corresponding parameters are presented in Table S3.

### 2.5. Method Performance

The analytical methods were validated following the SANTE guidance document (European Commission, 2019) [31] and NY/T 788–2018 [32], using linearity, the limit of detection (LOD), the limit of quantification (LOQ), accuracy, and precision. The LODs and LOQs for each metabolite were calculated by the minimum concentration with signal-to-noise (S/N) ratios of 3 and 10, respectively.

### 2.6. Homology Modeling of the Umami Receptor T1R1/T1R3 and Molecular Docking with Low Molecular Weight Compounds

Homology modeling of the umami receptor T1R1/T1R3 was conducted via Discovery Studio (DS, version 2.1; Neo Trident Technology Ltd.) according to previous reports [33]. The metabotropic glutamate receptor (PDB ID: 1EWK) was used as a template for homology modeling. Moreover, to verify the reliability of the selected homology model, a Ramachandran plot was used for evaluation. In addition to applying AlphaFold 2.1 for modeling (Figure S2), the calculated Ramachandran plot demonstrated that close to 99.3% of the residues were located in the allowed regions (89.3% of the residues in the most favored regions and 10.0% of the residues in the allowed regions), and only 0.7% of the residues were located in the disallowed regions. According to the critical evaluation principle of 90%, the conformation of homologous models with the metabotropic glutamate receptor as the template was reasonable.

The structure of the model needs to be optimized via energy minimization. The energy of the peptides was minimized via minimization and the CHARMM force field; that is, the energy minimization calculated via molecular mechanics was used to correct the unfavorable noncovalent bond contact, expansion bond geometry, and energy minimum state. To obtain a model closer to the real structure, the minimization module of Discovery Studio was used to minimize energy. In this study, the CHARMM force field was selected as the force field of the initial structure energy. The smart minimizer algorithm, with an energy minimization of 5000 steps, was revised and repeatedly optimized according to the molecular structure in operation and combined with the evaluation of the evaluation software; finally, the most reasonable and realistic structure was formed. Semiflexible docking of the receptor–ligand complex (CDOCKER) was conducted via Discovery Studio. The compound conformation of the receptor and ligand was assessed by the docking interaction energy, which was also used as the basis for ranking low molecular weight compounds.

### 2.7. Data Processing, Statistical Analysis, and Metabolic Pathway Analysis

The biomarkers between different groups were recognized by FC < 0.5 (downregulation) or FC > 2 (upregulation) with *p* < 0.05. Multivariate data analysis, including principal component analysis (PCA) and orthogonal partial least squares discriminant analysis (OPLS-DA), was performed via SIMCA-P 14.1 software (Umetrics, Umea, Sweden). Hierarchical clustering analysis (HCA) and heatmaps were constructed via Multi Experiment Viewer (MeV) software. One-way ANOVA was conducted via IBM SPSS Statistics 23 according to Duncan’s test (*p* < 0.05). Metabolomic pathway analysis of biomarkers with significant differences was performed via MetaboAnalyst 5.0 (https://www.metaboanalyst.ca) (accessed on 26 November 2022) on the basis of the KEGG metabolic pathway.

## 3. Results and Discussion

### 3.1. Optimization of the Extraction Step

**Figure 1:**
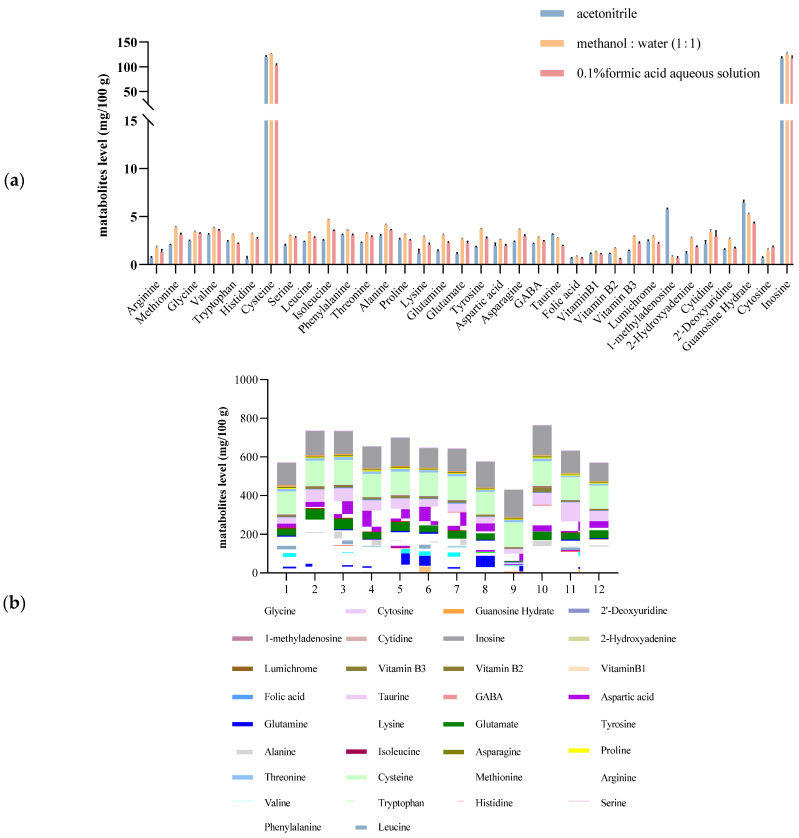
Effects of three typical extraction solvents on the extraction of metabolites (**a**); the Plackett–Burman design and the concentrations of typical metabolites (**b**).

The extraction solvent was optimized, and the extraction results were obtained from three typical extraction solvents, acetonitrile, 0.1% formic acid aqueous solution, and methanol/water = 1:1. The results revealed that methanol/water = 1:1 resulted in the optimal extraction efficiency. Figure 1 shows the effects of three typical extraction solvents on the extraction of metabolites.

The PB factorial design experiment was used to further optimize the pretreatment conditions. The chosen low-level (−1) values and high-level (1) values of extraction solvent volume (V), vortex time (t_1_), ultrasonic time (t_2_), ultrasonic temperature (T), and ultrasonic intensity (E) were 2 and 4 mL, 1 and 3 min, 10 and 20 min, 20 and 40 °C, and 60 and 100 W, respectively. The results of the PB design experiment indicated that the extraction solvent volume (V) and vortex time (t_1_) had significant effects on the extraction efficiency, and 2 mL and 3 min were determined to be the optimum extraction solvent volume and vortex time, respectively, through single-factor experiments. Finally, the ultrasonic temperature, time and power were determined to be 20 °C, 3 min and 60 W, respectively. PB design has been used to optimize multiple parameters that affect extraction efficiency. Li et al. [34] used aqueous alcohol as the extraction solvent to obtain high yields of flavonols and anthocyanins from blackcurrant via a PB design. Li et al. [35] investigated the factors affecting the high extraction yield of purines via PB design for screening.

### 3.2. Method Optimization and Quality Control

The total ion chromatograms of the 39 mixed metabolomics solutions are shown in Figure S1. The linear regression equation, correlation coefficient, linear range, limit of detection, limit of quantitation, and retention time of 34 nutrients and metabolites were obtained by repeated injection of a matrix-matched standard solution that included 12 gradients within 0.1–500 μg/L. The results are shown in Table S4. The LODs and LOQs of the 34 nutrients ranged from 0.01 to 5 μg/kg and 0.05 to 10 μg/kg, respectively.

### 3.3. Variations in Total Nutrient Metabolism during Storage

**Figure 2:**
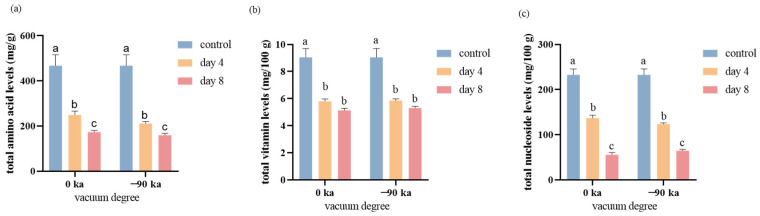
Changes in the concentrations of amino acids (**a**), vitamins (**b**), and nucleosides (**c**) after storage at 0 kPa and −90 kPa for 4 and 8 days. Bars with different letters (a, b, c) indicate significant differences between groups.

Initially, an analysis was conducted based on changes in odor and color at different sampling times. The fresh salmon purchased had a bright color and was accompanied by a fresh, sweet fragrance. During the experiment, it was observed that the muscle color of the salmon became increasingly pale over time. After 4 days of storage, the salmon stored at 0 °C still retained a faint pleasant aroma. By the 8th day of storage, the salmon stored at 0 °C had developed a slight fishy odor, with the pleasant fragrance had disappeared. On the basis of the established metabolomics method, the nutritional components of small molecules in salmon vacuum packaged at 0 kPa and −90 kPa and stored for 8 days were analyzed. The nutrients and metabolites present during storage can generally be divided into three groups according to the results of targeted metabolomics analysis. The changes in 34 metabolites, amino acids, vitamins, and nucleosides are shown in Figure 2.

The decreasing trends were most notable for vitamins and nucleosides. Overall, the impact of vacuum pressure on the concentrations of these three constituents were not significantly. The total amino acids in the group stored under −90 Kpa were observed to be lower in comparison to those in the 0 Kpa group. The total vitamin and nucleoside contents were 5.13 mg/100 g and 5.30 mg/100 g at 0 kPa and −90 kPa, respectively, after 8 days, which decreased by 43.33% and 41.42%, respectively. The total amino acid content was 173.27 mg/100 g and 159.55 mg/100 g under storage conditions of 0 kPa and −90 kPa after 8 days, which decreased by 62.95% and 65.89%, respectively.

### 3.4. Changes of Free Amino Acids during Storage

Salmon contains high amounts of free amino acids, which decrease significantly with increasing storage time. From 0 to 4 days, the total content decreased from 467.72 mg/100 g to 249.17 mg/100 g and 210.63 mg/100 g at 0 kPa and −90 kPa, representing decreases of 46.73% and 54.97%, respectively. From 4 to 8 days, the total content decreased from 249.17 mg/100 g to 173.27 mg/100 g at 0 kPa or from 210.63 mg/100 g to 159.55 mg/100 g at −90 kPa, corresponding to decreases of 30.46% and 24.25%, respectively. The relevant mechanisms might include the degeneration of fish myofibril protein during storage, such as changes in spatial structure, solubility, Ca^2+^-ATPase activity, sulfhydryl and disulfide bond content, and surface hydrophobicity, which lead to changes in amino acid content [36,37]. The activities of microorganisms and enzymes during storage [38].

However, the concentrations of some amino acids, including glycine, serine, GABA, taurine, and isoleucine, tended to differ; these compounds initially decreased on Day 4 but then increased on Day 8. This change probably resulted from their release from protein degradation by proteases [39]. As described above, the storage of salmon causes changes in amino acids, and these changes differ depending on the degree of vacuum and storage time. After 4 days of storage at 0 °C, the amino acid content in salmon decreased rapidly, possibly because of the degeneration of fish myofibril protein during storage, such as changes in spatial structure, solubility, Ca^2+^-ATPase activity, sulfhydryl and disulfide bond content, and surface hydrophobicity, which led to changes in amino acid content.

To evaluate the taste contribution of the individual taste compounds, 19 flavorful amino acids quantitatively determined in the salmon were grouped into five classes according to their taste qualities (Table 1). On the one hand, the umami-like taste sensation includes glutamic and aspartic acid. The glutamic acid exhibited an umami taste, differing in concentration from 7.90 mg/100 g to 4.56 mg/100 g after storage for 8 days at 0 °C/0 kPa. The aspartic acid concentration decreased from 3.08 mg/100 g to 0.82 mg/100 g after storage for 8 days at 0 °C/0 kPa. The total umami amino acid content decreased by 51.1% after storage for 8 days at 0 °C/0 kPa. On the other hand, alanine decreased significantly among the six kinds of sweet amino acids and was undetectable after 4 days of storage. Among the bitter-tasting compounds, the concentration of histidine was the highest, decreasing from 93.28 mg/100 g to 40.14 mg/100 g after storage for 8 days at 0 °C/0 kPa. It is noteworthy that bitter-tasting amino acids such as phenylalanine, tyrosine, isoleucine, and histidine exhibit significant variation under different storage conditions. Particularly, the content of histidine notably decreased to 35.07 mg/100 g after 8 days of storage at −90 kPa, which could substantially affect the bitterness level of the salmon. Furthermore, glutamine and aspartic acid, known as umami-tasting amino acids, also show a decline in their content after 8 days of storage at −90 kPa, potentially reducing the umami flavor of the salmon. Compared with the total content of sweet-tasting compounds and total content of bitter-tasting compounds, the total amount of sweet amino acids in fresh salmon is greater than that of bitter amino acids, but with increasing storage time, the content of bitter amino acids is greater than that of sweet amino acids, which may affect the taste of salmon. The compounds impart sour and/or mouth-drying sensations to the oral cavity, containing GABA and pyroglutamic acid [40]. Our results revealed that GABA tended to increase with increasing storage time. The increasing trend of GABA may be due to the conversion of glutamate to GABA by glutamate decarboxylase. The content of pyroglutamic acid slightly decreased with increasing storage time. Overall, the total content of sour-tasting and mouth-drying compounds was reduced by 34.1%. The total content of salt-tasting compounds decreased by 24.2% during the storage process.

### 3.5. Changes in Vitamins and Nucleosides during Storage

**Figure 3:**
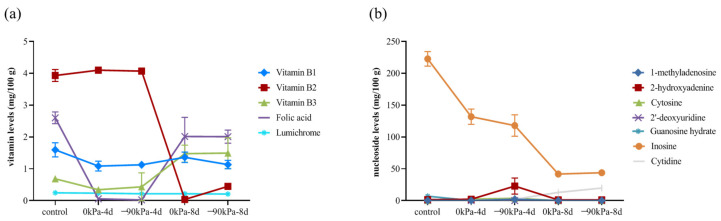
Changes in vitamin (**a**) and nucleoside (**b**) contents at different storage times and in the amount of vacuum used.

The total vitamin content in salmon clearly decreased during storage, ranging from 9.06 mg/100 g to 5.13 mg/100 g during storage at 0 kPa. High vitamin B1, folic acid, and vitamin B2 contents were found (Figure 3a). The vitamin B2 content varied from 3.93 mg/100 g to 0.45 mg/100 g after 8 days at −90 kPa. However, the vitamin B2 content was close to zero (0.033 mg/100 g) after 8 days at 0 kPa. A high vacuum degree is relatively beneficial to the maintenance of vitamin B2. Interestingly, the contents of vitamin B3 and folic acid increased at 8 days, whereas those of vitamin B3 and folic acid decreased first and then increased with increasing storage time, possibly because tryptophan can be converted into niacin [41]; folic acid is a combination of p-aminobenzoic acid, tretin, and glutamic acid [42]; and the consumption of amino acids (such as tryptophan and glutamic acid) during storage may promote the production of folic acid. Vitamin B3 and folic acid are also involved in the synthesis of purines and pyrimidines and the mutual conversion of amino acids.

Nucleosides are vital for the regulation of substance metabolism and biological functions in cells and are the second messengers of life information transmission. Purine metabolism is closely related to the metabolic response to temperature stress [43]. The concentrations of 2′-deoxyuridine, cytidine, 1-methyladenosine and guanosine hydrate decreased below the limits of detection after 4 days of storage. 2-Hydroxyadenine was also undetectable after 8 days of storage. There are more intense changes in nucleoside content within the first 4 days (Figure 3b). This could indicate that the metabolic activities of the salmon’s cells are more active during this period.

### 3.6. Unique Biomarkers for Salmon during Storage

**Figure 4:**
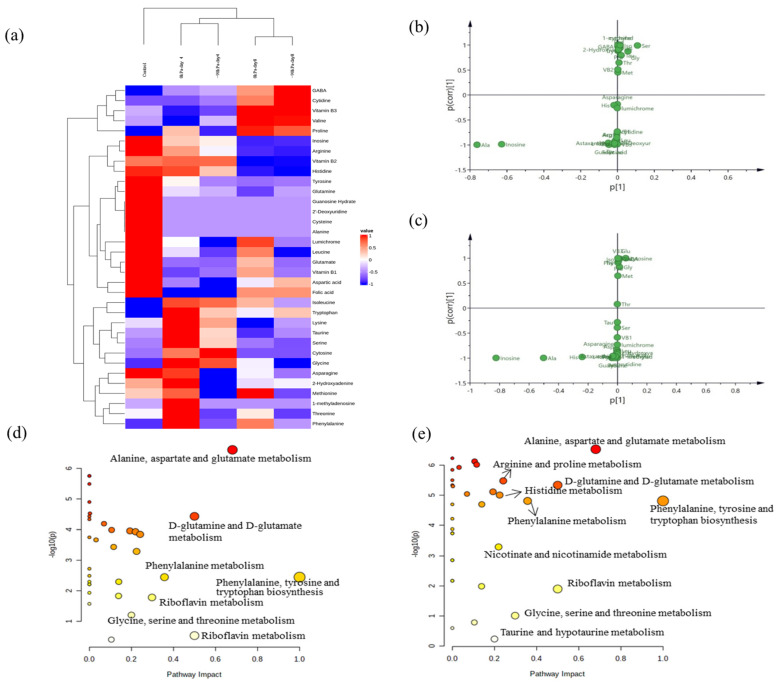
Heatmaps generated by hierarchical Pearson clustering for 34 nutrients in two different packing vacuums and storage times (**a**). S-plot of 34 nutrients obtained via PCA after storage for 4 days (**b**) and 8 days (**c**). KEGG pathway analysis showing changes in metabolism after storage for 4 days (**d**) and 8 days (**e**). Note: the size and color of the circle represent the pathway effect and pathway significance, respectively. The larger and more deeply colored circles in the upper right corner represent the pathways that had a strong effect with greater significance on salmon metabolism. (For interpretation of the references to color in this figure legend, the reader is referred to the web version of this article).

In the present study, multivariate data analysis was performed to profile and visualize the variation in salmon under different storage conditions, and the correlations between the metabolites and storage conditions were analyzed. First, five groups of samples are shown with heatmaps generated via hierarchical Pearson clustering on the basis of the average contents of 34 components (Figure 4a). The heatmaps show the contents of 34 nutritional compounds in salmon stored for different durations and under different degrees of vacuum packing. The cluster analysis on the basis of mean values revealed two major clusters on the basis of storing salmon for 4 days and 8 days. This means that the changes in 34 small molecule nutrients can distinguish between salmon stored for different periods of time under vacuum packaging conditions.

The S-plot chart revealed large changes in alanine and inosine contents after 4 days of storage, whereas greater changes in histidine contents were observed after 8 days of storage (Figure 4b,c). Therefore, alanine, inosine, and histidine are potential biomarkers associated with salmon freshness. Some previous studies reported similar results and identified histidine metabolism as a significantly enriched pathway correlated with chilled chicken spoilage [44,45].

**Figure 5:**
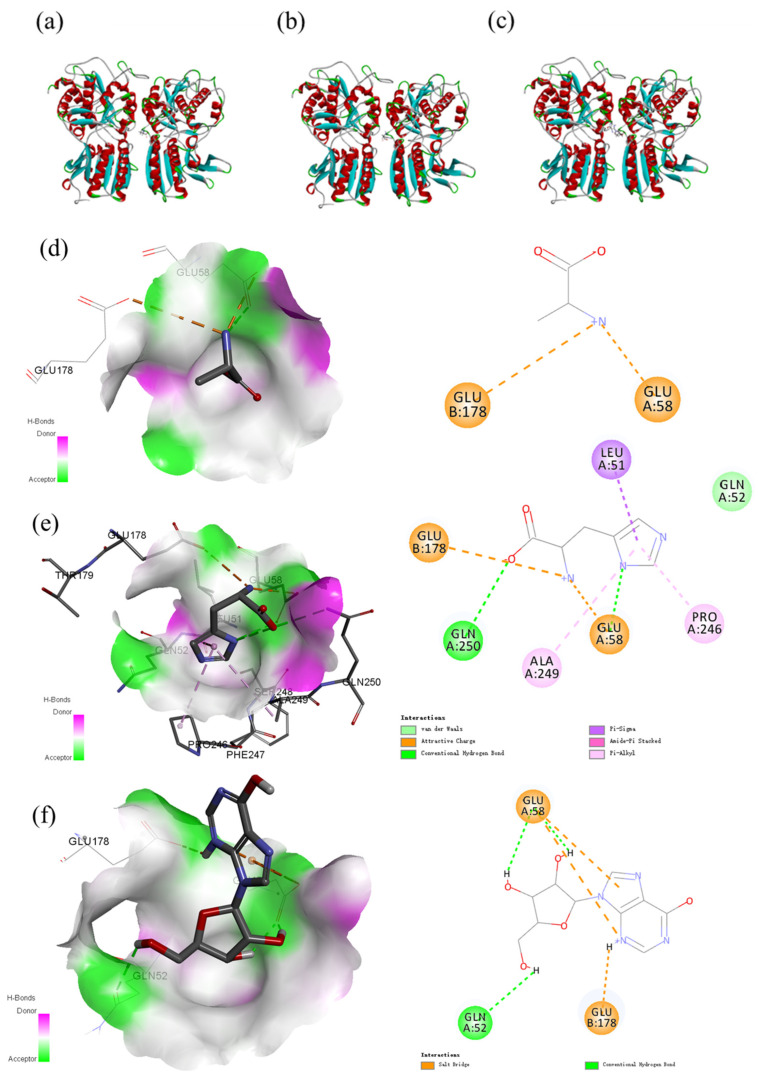
Docking results of different metabolites, alanine (**a**), histidine (**b**), and inosine (**c**), with T1R1/T1R3; the mechanism of the binding of alanine with T1R1/T1R3 (**d**); the mechanism of the binding of histidine with T1R1/T1R3 (**e**); and the mechanism of the binding of inosine with T1R1/T1R3 (**f**).

Umami and sweetness were the dominant tastes of salmon. Free amino acids are important tastants in food and display different taste properties, such as sweetness, umami, and bitterness. Researchers agree that amino acids and nucleotides, such as L-glutamic acid and 5′-ribonucleotides, are important for umami taste, thus stimulating umami taste [46]. It is not known whether other small molecules affect the flavor of salmon. Therefore, three small-molecule nutrients in salmon that vary dramatically with storage time were docked with umami receptors to assess the contribution of these small molecules to taste. The typical taste receptors T1R1-T1R3 can be activated by binding to several natural ligands. Here, we used homology modeling and molecular docking simulations to study the effects of three small-molecule nutrients, inosine, Ala, and His, on the structural dynamics of T1R1-T1R3 and to identify key residues involved in the recognition of binding ligands. The three representative dipeptides, inosine, alanine, and histidine, which can bind to different pockets of T1R1/T1R3, with the best pose of the energy interaction, are shown in Figure 5. The active amino acid residues play important roles in forming interactions, including Glu58, Glu178, Ala249, Pro246, and Leu51. Glu and Ala were the primary docking amino acids, which was similar to previous studies [33]. Analysis of protein–ligand docking of the model T1R1/T1R3 protein with inosine, alanine, and histidine revealed that T1R1/T1R3 binds to alanine with a relatively high binding affinity energy of −22.202 kcal/mol, alanine forms a salt bridge interaction with the GLU58 of the TIR1 protein, and alanine forms a salt bridge interaction with the GLU178 of the TIR3 protein, thus allowing the ligand to bind to the active pocket of the protein to form a complex. While histidine has a free binding energy of −27.792 kcal/mol, histidine can form salt bridges, hydrogen bonds, and conjugation interactions with protein binding pockets; form salt bridge interactions with GLU58 of the TIR1 protein; and form pi–alkyl–sigma conjugation interactions with Ala249, Pro246, and Leu51. It forms hydrogen bond interactions with Gln250 and Glu58, and histidine forms salt bridge interactions with Glu178 of the TIR3 protein. Inosine can effectively bind to the active pocket of a protein, and its binding energy is −37.1197 kcal/mol. Inosine can interact well with the protein binding pocket through salt bridges, hydrogen bonds and conjugation and form salt bridge interactions with GLU58 of the TIR1 protein. GLN542 and GLU58 form hydrogen bond interactions and salt bridge interactions with GLU178 of the TIR3 protein.

During salmon storage, 34 small-molecule metabolites were studied, three of which were selected, including Ala, his, and inosine, and the molecular docking results revealed that it could bind to the typical umami receptor TIR1/TIR3. These findings are consistent with our results, and the sweet amino acid Ala is widely recognized as one of the key factors in the development of the characteristic sweetness of seafood because of its high degree of binding with TIR31/TIR3 [47,48]. Our results revealed that the binding strength of histidine and inosine to the typical umami receptor TIR1/TIR3 was greater than that of Ala. Therefore, Ala, inosine, and histidine can be used as key indices of the freshness of salmon during storage.

### 3.7. Changes in Metabolic Pathways Associated with Different Packages and Durations of Salmon According to Targeted Profiling as Metabolite Growth Occurs

As mentioned above, the metabolites of salmon changed strongly after storage for 4 days and 8 days. Therefore, the peak intensities of different metabolic pathways on Days 4 and 8 of 0 kPa packing vacuum for salmon were submitted to MetaboAnalyst to analyze the major metabolic pathways that occurred after storage for 8 days (Figure 4d,e). Amino acid metabolism—including alanine, aspartate, and glutamate metabolism; phenylalanine, tyrosine, and tryptophan biosynthesis; D-glutamine and D-glutamate metabolism; phenylalanine metabolism; glycine, serine, and threonine metabolism; and riboflavin metabolism—plays important roles in the salmon storage process. Additionally, there were differences in the metabolic pathways among the different storage times. Notably, nicotinate and nicotinamide metabolism, taurine and hypotaurine metabolism, arginine and proline metabolism, and histidine metabolism pathways were significantly affected in the samples stored for 8 days compared with those stored for 4 days.

At the end of the experiment, fleshy corruption was observed, which may be related to alanine, aspartate, and glutamate metabolism; phenylalanine, tyrosine, and tryptophan biosynthesis; and D-glutamine and D-glutamate metabolism degradation. This finding is consistent with previous findings in seafood [49]. Shumilina et al. [50] reported that salmon thawing caused a significant increase in the concentration of phenylalanine in stored salmon muscle, and the concentration of phenylalanine also constantly increased during fresh storage. Zhang et al. (2021) similarly reported spoilage in chilled chicken, and the differential metabolism-related pathways were enriched primarily in amino acids such as phenylalanine, tyrosine and tryptophan biosynthesis, and phenylalanine metabolism [23].

## 4. Conclusions

In this work, the extraction method for 34 nutrients from salmon was optimized via the Plackett–Burman (PB) test. A sensitive and rapid targeted metabolomics method for the simultaneous determination of 34 nutrients was successfully established via ultra-performance liquid chromatography–triple quadrupole/linear ion trap composite mass spectrometry (UHPLC-QTRAP/MS), and 34 nutrients stored at 0 °C under different vacuum conditions (0 kPa or −90 kPa) were varied after 4 and 8 days. In summary, the levels of 34 metabolites in salmon species varied greatly with increasing storage time. The packaging conditions at 0 °C and 0 kPa are more suitable for the preservation of most nutrients in salmon. After 8 days of storage, the content of free amino acids and vitamins decreased by nearly half, and the content of bitter compounds in the free amino acids increased. After 4 and 8 days of storage, the metabolism of alanine, aspartic acid, and glutamate; the biosynthesis of phenylalanine, tyrosine, and tryptophan; and the metabolism of D-glutamine and D-glutamate were the three main changes during storage. More specifically, inosine, alanine, and histidine, whose levels changed significantly, can bind to the typical umami receptor TIR1/TIR3 and can be valuable for the measurement of differentially abundant metabolites to monitor and determine the freshness or spoilage stage of salmon.

## Figures and Tables

**Table 1:** Analysis and comparison of 19 kinds of flavor amino acids in salmon.

Tastant	Compound	Content (mg/100 g)				
		Control	0 kPa-4d	0 kPa-8d	−90 kPa-4d	−90 kPa-8d
**Umami-tasting**	Glutamine	7.90 ± 0.14	4.86 ± 0.41	4.56 ± 0.11	3.78 ± 0.43	4.45 ± 0.5
	Aspartic acid	3.08 ± 0.48	1.35 ± 0.18	0.82 ± 0.13	1.74 ± 0.11	2.13 ± 0.17
**Umami-tasting compounds total content**		**10.99** **± 0.62**	**6.21** **± 0.59**	**5.37** **± 0.24**	**5.52** **± 0.54**	**6.58** **± 0.67**
Sweet-tasting	Glycine	19.45 ± 1.25	27.68 ± 3.57	26.35 ± 1.34	22.75 ± 1.29	18.85 ± 1.49
	Serine	5.52 ± 0.31	21.35 ± 2.14	11.22 ± 2	5.25 ± 0.36	4.04 ± 0.11
	Threonine	13.03 ± 0.81	14.24 ± 0.64	12.45 ± 0.48	13.14 ± 1.04	12.44 ± 0.16
	Proline	4.37 ± 0.22	5.28 ± 0.28	4.51 ± 0.14	5.67 ± 0.33	5.58 ± 0.48
	Lysine	3.38 ± 0.25	5.25 ± 0.57	4.14 ± 0.22	2.06 ± 0.13	3.06 ± 0.66
	Alanine	110.44 ± 7.02	NG	NG	NG	NG
**Sweet-tasting compounds total content**		**156.19** **± 9.86**	**73.79** **± 7.2**	**58.67** **± 4.18**	**48.88** **± 3.13**	**43.97** **± 2.91**
Bitter-tasting	Arginine	2.31 ± 0.34	1.66 ± 0.11	1.26 ± 0.14	0.64 ± 0.05	0.61 ± 0.11
	Valine	5.75 ± 0.14	3.7 ± 0.66	6.04 ± 0.55	9.15 ± 0.43	9.06 ± 0.36
	Tryptophan	1.31 ± 0.04	1.75 ± 0.2	1.62 ± 0.04	1.56 ± 0.09	1.62 ± 0.11
	Leucine	4.26 ± 0.13	3.58 ± 0.22	3.27 ± 0.23	3.94 ± 0.05	3.20 ± 0.14
	Phenylalanine	6.31 ± 0.18	7.54 ± 0.39	6.39 ± 0.27	7.13 ± 0.18	6.55 ± 0.32
	Tyrosine	9.05 ± 0.37	3.23 ± 0.98	1.13 ± 0.15	0.81 ± 0.13	0.96 ± 0.02
	Isoleucine	2.53 ± 0.13	4.70 ± 0.04	4.27 ± 0.18	4.60 ± 0.31	3.53 ± 0.66
	Histidine	93.28 ± 8.13	90.52 ± 5.23	40.14 ± 2.03	75.06 ± 1.63	35.07 ± 1.92
**Bitter-tasting compounds total content**		**124.8 ± 9.46**	**116.67** **± 7.83**	**64.11** **± 3.59**	**102.88** **± 2.87**	**60.61** **± 3.66**
Salty-tasting	Methionine	2.64 ± 0.14	2.86 ± 0.34	2.00 ± 0.07	2.96 ± 0.28	2.21 ± 0.11
**Salty-tasting compounds total content**		**2.64 ± 0.14**	**2.86 ± 0.34**	**2.00** **± 0.07**	**2.96** **± 0.28**	**2.21** **± 0.11**
Sour-tasting and mouth-drying	GABA	0.27 ± 0.001	0.69 ± 0.1	0.77 ± 0.03	1.37 ± 0.13	1.99 ± 0.19
	Pyroglutamic acid	12.19 ± 0.37	9.68 ± 0.32	7.44 ± 1.11	8.61 ± 0.39	8.82 ± 0.5
**Sour-tasting and mouth-drying compounds total content**		**12.46 ± 0.37**	**10.37 ± 0.41**	**8.21** **± 1.13**	**9.98** **± 0.51**	**10.81** **± 0.69**

NG: not detected.

## Data Availability

The original contributions presented in the study are included in the article/Supplementary Material, further inquiries can be directed to the corresponding authors.

## Supplementary Materials

The following supporting information can be downloaded at: https://www.mdpi.com/article/10.3390/foods13172714/s1. Figure S1: Total Ion Chromatogram of the 34 mixed solution of metabolomics (100 μg/L); Figure S2: Structure of T1R1/T1R3 with the metabotropic glutamate receptor (PDB ID: 1EWK) as the template (A) and Ramachandran Plot (B); Table S1: Experimental design of salmon packaging; Table S2: Five-factor Plackett Burman (PB) test design; Table S3 The MS information of 34 nutrients in salmon; Table S4 Linearity range and equation, determination coefficient (R2), limit of detection, limit of quantification of 34 metabolisms.
