# Supplementary material for: Optimization and Detection of Freshness Biomarkers of Atlantic Salmon Subjected to Different Vacuum Packaging Conditions during Storage at 0 °C by Metabolomics and Molecular Docking
Source: Foods. 2024 Aug 27;13(17):2714. doi: 10.3390/foods13172714 (PMC11394979; doi:10.3390/foods13172714)
Supplement: Supplementary file 1 [file foods-13-02714-s001.zip › foods-3130349-supplementary.pdf]

List of contents

Table S1. Experimental design of salmon packaging

Table S2. Five-factor Plackett Burman (PB) test design

Table S3. The MS information of 34 nutrients in salmon

Table S4. Linearity range and equation, determination coefficient (R2), limit of detection, limit of quantification of 34 metabolisms

Figure S1. Total Ion Chromatogram of the 34 mixed solution of metabolomics (100 μg/L)

Figure S2. Structure of T1R1/T1R3 with the metabotropic glutamate receptor (PDB ID: 1EWK) as the template (A) and Ramachandran Plot (B)

Table S1. Experimental design of salmon packaging

vacuum degree（kPa）	storage temperature（℃）	storage time（day）
0	0	4, 8
-90	0	4, 8

Table S2. Five-factor Plackett Burman (PB) test design

Number	V	t_1	t_2	T	E
1	1	1	-1	1	1
2	1	-1	-1	-1	1
3	-1	1	1	-1	1
4	1	1	-1	-1	-1
5	1	-1	1	1	-1
6	1	1	1	-1	-1
7	-1	1	-1	1	1
8	-1	1	1	1	-1
9	-1	-1	1	-1	1
10	1	-1	1	1	1
11	-1	-1	-1	1	-1
12	-1	-1	-1	-1	-1

Table S3. The MS information of 34 nutrients in salmon

No.	Metabolites	RT min	Q1 Mass (Da)	Q3 Mass (Da)	DP/V	CE/V
1	Arginine	2.82	175.2	70	50	40
				60	28	18
2	Methionine	4.80	150.2	133	46	15
				104	39	15
3	Glycine	2.89	76.2	30	29	15.2
4	Valine	4.50	118.1	72.2	55	14
5	Tryptophan	8.04	205.1	146	27	25
6	Histidine	2.80	156.1	100.1	41	20
				82.9	30	32
7	Serine	2.90	106.1	60	20	14
				70	22	14
8	Leucine	6.92	132.1	86.1	23.53	14
9	Isoleucine	6.71	132.1	69.1	41	24
10	Phenylalanine	6.71	166.1	120.1	35	19
				103	40	37
11	Threonine	2.99	120.1	74.1	20	14
				56	31	20
12	Alanine	2.97	90.1	44	40	14
13	Proline	3.22	116.1	70	31	21
14	Lysine	2.94	147.1	130	25	14
				84.1	19	14
15	Glutamine	2.94	147.1	130	20	14
				84	24	21
16	Glutamate	3.00	148.2	102	20	14
				84.1	17	22
17	Tyrosine	5.93	182.1	165	40	13.7
				136.1	58	19.3
18	Aspatic acid	2.93	133.9	88	40	14.25
				74.3	31	17.9
19	Asparagine	6.92	133.1	87	40	14.8
				74	30	21.1
20	γ-aminobutyric acid	3.04	104.1	87	26	13.2
				69	26	21
21	Pyroglutamic acid	2.95	130.1	84.1	45	18
				56.2	40	29
22	Taurine	2.93	124	80	-50	-27
				107	-65	-21
23	Folic acid	7.63	442.2	295.2	25	20
				176	25	40
24	Thiamine	8.17	377.3	243	16	32
				176.1	16	50
25	Riboflavin	8.31	265.2	122.1	19	20
				144.2	14	20
26	Lumichrome	9.97	243.2	198	80	30
				172	130	30
27	Cytidine	3.35	244.2	172	130	30
28	Inosine	6.57	267	107.9	-40	-55
29	Guanosine Hydrate	6.55	283.2	133	-60	-45
				108	-60	-45
30	1-methyladenosine	6.80	282.2	150	40	25
31	2'-Deoxyuridine	8.90	227	94.2	-50	-25
				111	-30	-15
32	Cytosine	6.54	112	95	80	25
				52.1	80	45
33	Adenine	3.04	133.9	65	-50	-40
				92	-50	-25
34	2-Hydroxyadenine	3.37	152	135	60	25
				107	170	46

Table S4. Linearity range and equation, determination coefficient (R2), limit of detection, limit of quantification of 34 metabolisms

NO.	metabolisms	Linear equation	R^2	LOD (μg/L)	LOQ(μg/L)
1	Arginine	$Y=9.78\times10^4X$	0.9915	0.1	0.5
2	Methionine	$Y=6.26\times10^4X+1.38\times10^5$	0.9993	0.05	0.2
3	Glycine	$Y=2.17\times10^3X$	0.9911	0.05	0.2
4	Valine	$Y=1.51\times10^5X+4.25\times10^6$	0.9968	0.1	0.2
5	Tryptophan	$Y=2.67\times10^5X+5.07\times10^4$	0.9999	0.01	0.05
6	Histidine	$Y=1.76\times10^5X$	0.9946	0.01	0.05
7	Serine	$Y=4.32\times10^4X$	0.9938	0.1	0.5
8	Leucine	$Y=8.19\times10^5X+3.31\times10^6$	0.9990	0.1	0.5
9	Isoleucine	$Y=5.20\times10^4X+7.80\times10^4$	0.9998	0.05	0.2
10	Phenylalanine	$Y=5.95\times10^5X+2.33\times10^6$	0.9992	0.05	0.2
11	Threonine	$Y=4.39\times10^{4X}+6.01\times10^5$	0.9921	0.1	0.2
12	Alanine	$Y=2.10\times10^4X+3.07\times10^5$	0.9938	0.01	0.05
13	Proline	$Y=2.59\times10^5X+2.30\times10^6$	0.9961	0.01	0.05
14	Lysine	$Y=1.21\times10^5X$	0.9965	5	10
15	Glutamine	$Y=9.42\times10^4X+1.11\times10^6$	0.9927	0.1	0.5
16	Glutamate	$Y=9.40\times10^4X+1.37\times10^6$	0.9920	0.1	0.5
17	Tyrosine	$Y=7.91\times10^4X+3.94\times10^5$	0.9971	0.05	0.2
18	Aspatic acid	$Y=3.58\times10^4X+3.60\times10^5$	0.9971	0.05	0.2
19	Asparagine	$Y=4.77\times10^4X+3.46\times10^5$	0.9966	0.1	0.2
20	γ-aminobutyric acid	$Y=5.59\times10^4X+6.74\times10^5$	0.9929	0.01	0.05
21	Taurine	$Y=2.60\times10^4X+3.18\times10^5$	0.9912	0.05	10
22	Folic acid	$Y=4.63\times10^4X+3.94\times10^5$	0.9925	0.05	0.5
23	Thiamine	$Y=8.93\times10^3X+8.21\times10^4$	0.9960	0.01	0.5
24	Riboflavin	$Y=2.13\times10^5X+2.55\times10^6$	0.9968	0.01	0.2
25	Vitamin A	$Y=2.13\times10^5X+2.55\times10^6$	0.9968	0.01	0.05
26	Cytidine	$Y=1.21\times10^5X+8.46\times10^5$	0.9968	5	10
27	Inosine	$Y=5.59\times10^3X+2.74\times10^4$	0.9964	0.1	0.5
28	Lumichrome	$Y=6.70\times10^5X+6.47\times10^6$	0.9947	0.05	0.2
29	Guanosine Hydrate	$Y=9.54\times10^2X+6.38\times10^3$	0.9962	0.05	0.2
30	1-methyladenosine	$Y=2.38\times10^6X$	0.9994	0.1	0.2
31	2'-Deoxyuridine	$Y=1.21\times10^5X+8.46\times10^5$	0.9966	0.01	0.05
32	Cytosine	$Y=1.82\times10^5X+1.26\times10^6$	0.9964	0.01	0.05
33	Adenine	$Y=3.28\times10^5X$	0.9915	5	10
34	2-Hydroxyadenine	$Y=1.04\times10^3X+3.02\times10^3$	0.9975	0.1	0.5

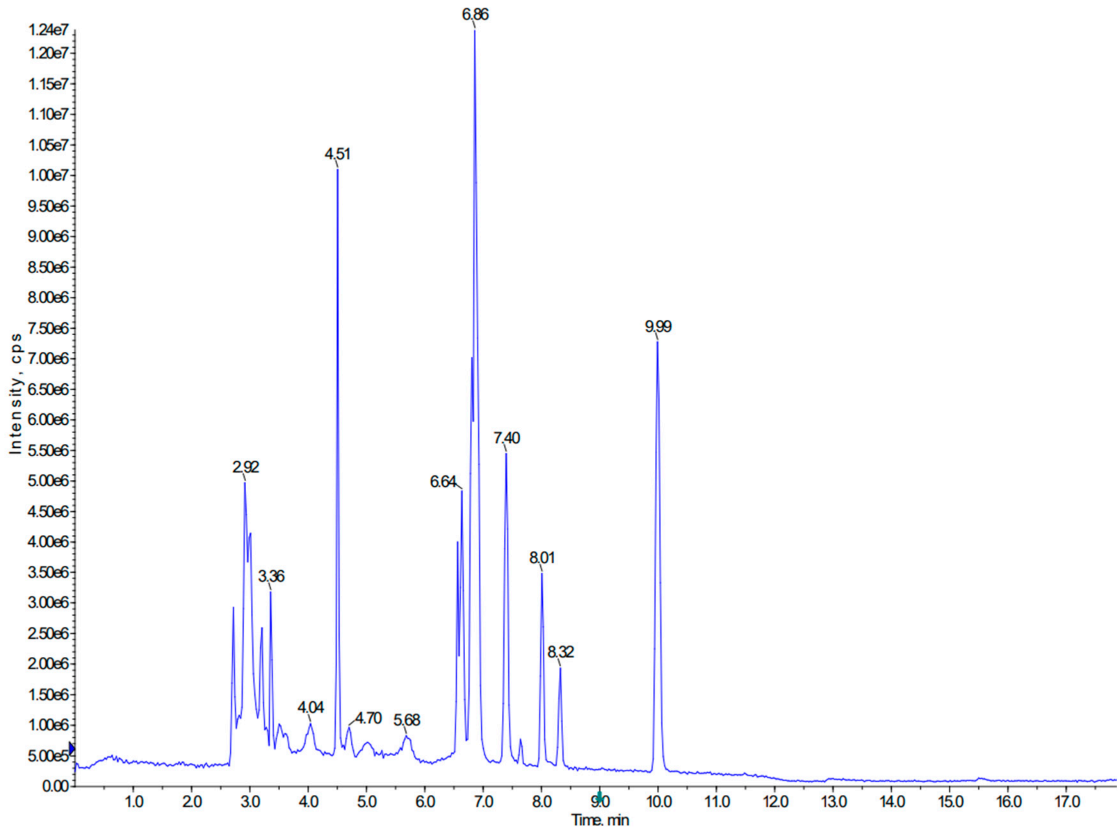

Figure S1. Total Ion Chromatogram of the 34 mixed solution of metabolomics (100 μg/L)

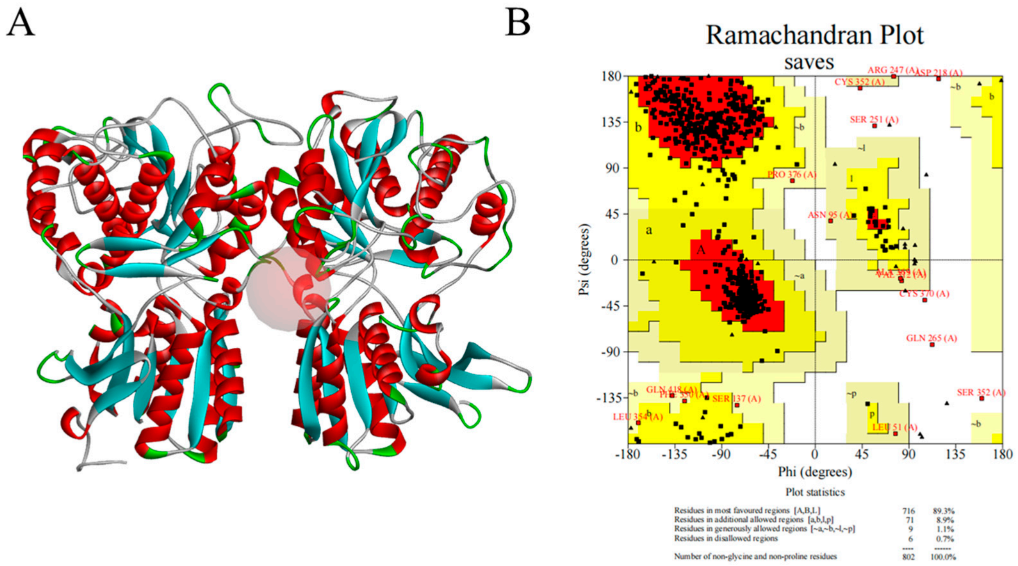

Figure S2. Structure of T1R1/T1R3 with the metabotropic glutamate receptor (PDB ID: 1EWK) as the template (A) and Ramachandran Plot (B)
